# Supplementary material for: Towards Characterization of Hass Avocado Peel and Pulp Proteome during Postharvest Shelf Life
Source: Proteomes. 2024 Sep 28;12(4):28. doi: 10.3390/proteomes12040028 (PMC11503343; doi:10.3390/proteomes12040028)
Supplement: Supplementary file 1 [file proteomes-12-00028-s001.zip › Supporting Information.pdf]

Supporting Information

ACC and ABA extraction

100 mg of fresh tissues were grounded to a fine powder using a mortar with a pestle and liquid nitrogen. The sample was dissolved in 1 mL of the extraction buffer containing methanol, isopropanol, and acetonitrile (20:79:1, v/v). The mix was homogenized with an ultrasonic bath (Cole Parmer, EW-08895-99) for 20 min at 4 °C and centrifuged at 10,000 x *g* for 15 min at 4 °C. Later, the supernatant was recovered, and the pellet washed twice with 0.5 ml of extraction buffer. The supernatants were pooled and dry with a rotary evaporator (Büchi, Rotavapor R-100). Dry samples were dissolved in 0.3 ml of methanol with 0.1% formic acid and filtered with 0.2 µm polytetrafluoroethylene membranes and transferred to 1.5 ml vials with 0.3 ml inserts.

ACC quantification by UPLC-MS/MS

ACC analysis was carried out with a 1290 infinity Agilent ultrahigh resolution liquid chromatography coupled to a 6460 Agilent triple quadrupole mass spectrometer. The column used in this study was an Agilent, Zorbax SB-C18, 2.1x50 mm, 1.8 Microns. The conditions of sample injection comprised a column temperature of 40 °C, 1 µL injection volume and a flow rate of 0.1 mL/min. The mobile phases included water with 0.1% of formic acid (A) and acetonitrile with 0.1% formic acid (B). The gradient elution profile was: starting from 5% solvent B, 5-80% solvent B within 12 min, isocratic 80% solvent B in 2 min and 80-5% solvent B for 1 min. The mass spectrometry conditions were set as follow: Gas Temperature, 300 °C; Gas Flow, 10 L/min; Nebulizer, 45 psi; Sheath Gas Temperature, 350 °C; Sheath Gas Flow, 11 L/min; Capillary voltage (positive and negative), 3,500 V and Nozzle voltage in

positive, 500 V. The fragmentor voltage was 100 V, and the cell accelerator voltage was 7 V for each compound. Besides, the calibration curve for ACC compound in a concentration range of 1 to 9 μM showed r^2 value of 0.99.

ABA quantification by UPLC-MS/MS

ABA analysis was carried out with a Class I Waters ultrahigh resolution liquid chromatography coupled to a Synapt G2-Si Waters quadrupole-time of flight mass spectrometer. The chromatography was carried out on an Acquity BEH column (1.7 μm, 2.1 x 50 mm) with a column and sample temperatures of 40 and 15 °C, respectively. The mobile phase consisted of (A) water and (B) acetonitrile, both with 0.1% of formic acid (SIGMA). The gradient conditions of the mobile phases were 0-1 min isocratic at 1% of B, 1-15 min linear gradient from 1 to 95% of B, 15-16 min isocratic at 95% of B, 16-17 min linear gradient from 95 to 1% of B, 17-20 min isocratic at 1% of B (total run time 20 min). The flow rate was 0.1 mL/min and 1 μL of extract was injected. The mass spectrometric analysis was performed with an electrospray ionization source in negative mode with a capillary, sampling cone and source offset voltages of 3,000, 40 and 80 V, respectively. The source temperature was 100° C and the desolvation temperature was 20 °C. The desolvation gas flow was 600 L/h and the nebulizer pressure was 6.5 Bar. Leucine-enkephalin was used as the lock mass (554.2615, $[M-H]^-$). The transition used for ABA quantification was 263.1283>153.0914. The data were acquired and processed with MassLynx (version 4.1) and TargetLynx (version 4.1). For ABA quantification a calibration curve was constructed with the next concentrations points: 0.5, 1, 2, 4, 6, 8, 10, 14, 16 and 18 μM. Each point was injected twice and the corresponding area was considered to perform the calibration curve. It was used a 2nd order curve regression with a coefficient of determination of 0.99.

2.4 Protein extraction, reduction, alkylation, and digestion

Phenol-based protein extraction was performed according to a previously described protocol. In brief, three grams of peel from five fruits were ground to a fine powder in liquid nitrogen using a mortar and pestle in the presence of 0.3 g of polyvinyl polypyrrolidone (PVPP). The powder was suspended in three volumes of ice-cold extraction buffer [(150 mM Tris-HCl, pH 8.0, 100 mM KCl, 1.4 M sucrose, 1% Triton X-100, 1% dithiothreitol (DTT)] enriched with 7.5 µL -Mercaptoethanol, 150 µL 100 mM phenylmethylsulfonyl fluoride (PMSF) per 15 mL of buffer, and 100 L· g-1 protease inhibitor cocktail (Sigma-Aldrich®, Cat No P9599)]. The samples were mixed, then an equal volume of phenol solution was added and vortexed vigorously. After shaking the samples on ice for 30 minutes, they were centrifuged at 10,000 x g for 30 minutes at 4 °C. The upper phenolic phase was precipitated overnight at -20 °C using five volumes of cold acetone enriched with 0.07% mercaptoethanol. Proteins were centrifuged at 3,000 x g for 30 minutes at 4 °C, and then the pellets were washed twice with ice-cold acetone. After drying for 5 minutes, the pellets were dissolved in 300 µL of solubilization buffer (50 mM phosphate buffer saline, pH 7.4, 1% SDS). The supernatant was recovered and centrifuged at 15,000 x g for 10 minutes at room temperature, and the soluble phase was recovered and stored at -80 °C. A BCA protein assay kit (Pierce, Thermo Scientific®, Cat No. 23225) was used to determine the protein concentration of each sample (Table 1). Proteins were separated by SDS-PAGE under denaturing conditions using conventional techniques, and they were stained with Sypro Ruby solution (Supelco, Cat No. S4942).

Table 1. Protein concentration of each sample analyzed in our study

	PEEL (MG/μL)	SD	PULP (MG/μL)	SD
**T0**	6.15411826	0.55548205	7.67272878	1.36055495
**T3**	7.56267997	1.33065286	7.3978575	1.82553967
**T6**	6.9490989	1.51590166	5.76856004	1.13197914
**T9**	6.9788874	1.53685399	6.41234845	1.44893215

We used 100 μg of protein extract in a final volume of 100 μL from each sample and extraction method for further analysis. These proteins were reduced for 45 minutes at 60 °C with 10 mM Tris (2-carboxyethyl) phosphine (TCEP) and alkylated for one hour at room temperature in darkness with 30 mM iodoacetamide (IA). After quenching the reaction with 30 mM DTT for 10 minutes, proteins were precipitated overnight at -20 °C in 1 mL of cold acetone. Each mixture was then centrifuged at 10,000 x *g* for 15 minutes at 4 °C, and the pellets were dried. Pellets were sonicated for 5 min minutes in 150 μL of 50 mM triethylammonium bicarbonate (TEAB) containing 0.1% SDS. Proteins were digested overnight at 37 °C with trypsin (Promega, Cat. No. Trypsin Gold, Mass Spectrometry Grade) at a 1:30 w/w trypsin:protein ratio. Then, trypsin was again added at a 1:60 w/w ratio of trypsin:protein for 4 hours at 37 °C to complete trypsinization.
